# A Case of Puerperal Septicemia

**Published:** 1880-10

**Authors:** Frederick Peterson


### A CASE OF PUERPERAL SEPTICEMIA.

BY FREDERICK PETERSON, M. D.

B. W., seamstress, aged 19, entered the Buffalo General Hospital, private room No. 7, Nov. 11, 1879, pregnant about five and one-half months, primipara. Until her confinement was very melancholy, spending one-half the time in weeping over her misfortunes. According to Fordyce Barker, this would be an important factor in complicating her troubles.

March 5.—A patient in room above her was attacked with idiopathic erysipelas of a most alarming nature, ending fatally in six days. As soon as the character of this disease was determined, our pregnant patient was removed to the pavilion, a wooden, well-ventilated and thoroughly disinfected building separate from the Hospital proper. All communication that in any way might prove infectious, was cut off from the main building.

March 11.—Labor began at 10 P. M; fiirst stage, six hours; second, three hours; third, five minutes; R. O. P. ten pound, male; perineum torn some distance into sphincter ani; put in three silk stitches immediately; later gave morphia gr. ⅛ because of pain and sleeplessness.

March 12.—Passed catheter toward evening, and continued to do so two or three times daily for several days; all right until

March 14.—7 P. M.: Pulse 153, temp. 102¾°, resp. 30; lochia offensive, tympanites; uterus enlarged, tender and extending to umbilicus; temp. through night 104°, and once 105°.

March 15, A. M.—Temp. 103°, pulse 120; breasts being full, and mother refusing to nurse child, evacuated them with pump and strapped with emp. belladonna; had no further difficulty with them; ordered vaginal douche: warm water Oj, acid. carbolic. ʒiii, and few grains pot. permang., night and morning. For two or three days used tinct. verat. vir. every three or four hours to lower pulse which had the desired effect; quinia, first in v gr.-doses, then in ji ss gr.-doses, with pulv. opii gr. i, given every four hours; turpentine stupes applied to abdomen; in two or three days metritis subsided; uterus returning to normal dimensions; stimulants given.

March 24.—Hard chill at midnight; followed by nausea and vomiting this morning, and pulse 154, temp. 105° C. T.

March 25, A. M.—Every symptom of collapse; brandy ammonia carb. given often in small quantities. Drs. White and Diehl saw the patient in this state, and death within a few hours was naturally prognosticated. Dr. W. advised the hypodermic injection of sulphuric ether as a last resort, but the patient in a short time began to rally, and the ether was not needed.

March 29.—Great tenderness in right iliac region, and a tumor size of fist has appeared just above Poupart's ligament. There is also a tumefaction of the right wall of vagina, making it difficult to introduce the nozzle of the syringe used in giving the douche. It was called a cellular abscess, but as fluctuation could not be obtained, it was thought best not to open it to-day; lochia scanty and purulent.

March 30.—Tumor suddenly disappeared and diarrhœa set in; pus and fæces pass through vagina and rectum. It seems that the abscess has burst into both rectum and vagina at the same time, making a fistula by the approximation of its walls; temp. now 100¼°; takes brandy and broken ice constantly; diarrhœa ceased the next evening.

April 7.—Bowels constipated for a week; sits up in bed; pulse and temp. nearly normal; no vaginal discharge; has had small abscesses on lips and trunk, one in right external auditory meatus and one on end of nose; knees very tender.

April 8.—Bowels moved; hard walls of abscess can be felt through abdomen, but has no tendency to refill; pulse dichrotic; feels smart but is very weak; for ten days following this date; had a typhoid temperature, 99°–100° morning, 102½-103¼° evening.

April 28.—Temp. and pulse normal; can sit up in a chair and walk a few paces; recto-vaginal fistula healed some time ago.

May 19, 1880.—Left hospital entirely well.

